# Physiological and Molecular Characterization of the Differential Response of Broccoli (*Brassica oleracea* var. *Italica*) Cultivars Reveals Limiting Factors for Broccoli Tolerance to Drought Stress

**DOI:** 10.1021/acs.jafc.1c03421

**Published:** 2021-08-27

**Authors:** Sergio Chevilly, Laura Dolz-Edo, José M. López-Nicolás, Luna Morcillo, Alberto Vilagrosa, Lynne Yenush, José M. Mulet

**Affiliations:** † Instituto de Biología Molecular y Celular de Plantas, Universitat Politècnica de València-Consejo Superior de Investigaciones Científicas, 46022 Valencia, Spain; ‡ Departamento de Bioquímica y Biología Molecular-A, Facultad de Biología, Universidad de Murcia, 30100 Murcia, Spain; § Fundación Centro de Estudios Ambientales del Mediterráneo, Joint Research Unit University of Alicante—CEAM, University of Alicante, 03080 Alicante, Spain

**Keywords:** broccoli, drought stress, plant hormones, essential amino acids, primary metabolites

## Abstract

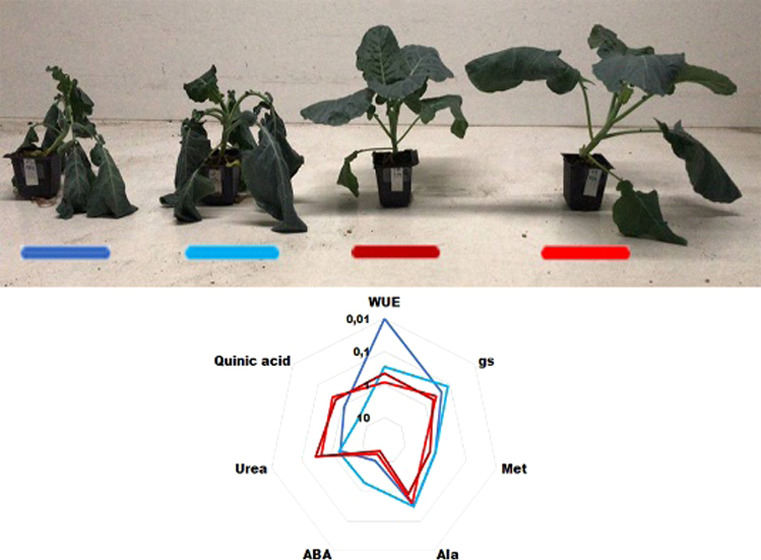

Broccoli is a cruciferous crop rich in health-promoting metabolites. Due to several factors, including anthropogenic global warming, aridity is increasing in many cultivation areas. There is a great demand to characterize the drought response of broccoli and use this knowledge to develop new cultivars able to maintain yield under water constraints. The aim of this study is to characterize the drought response at the physiological and molecular level of different broccoli (*Brassica oleracea L*. var. *Italica* Plenck) cultivars, previously characterized as drought-sensitive or drought-tolerant. This approach aims to identify different traits, which can constitute limiting factors for drought stress tolerance in broccoli. For this purpose, we have compared several physiological parameters and the complete profiles of amino acids, primary metabolites, hormones, and ions of drought-tolerant and drought-sensitive cultivars under stress and control conditions. We have found that drought-tolerant cultivars presented higher levels of methionine and abscisic acid and lower amounts of urea, quinic acid, and the gluconic acid lactone. Interestingly, we have also found that a drought treatment increases the levels of most essential amino acids in leaves and in florets. Our results have established physiological and molecular traits useful as distinctive markers to predict drought tolerance in broccoli or which could be reliably used for breeding new cultivars adapted to water scarcity. We have also found that a drought treatment increases the content of essential amino acids in broccoli.

## Introduction

The human population is currently above seven billion and is expected to increase to nine billion in 2050. To provide a robust food supply for this growing population, agricultural productivity must increase concomitantly. In the current context of anthropogenic global warming and increasing temperatures and CO_2_ concentrations in the atmosphere, the precipitation regimes are changing, increasing aridity and limiting the amount of water available for agriculture.^1^ Drought affects millions of people per year and is considered one of the main causes of famines. Breeding has improved the effectiveness of some crops under drought stress.^2^ However, there is still a need to develop novel cultivars of crops able to grow in arid lands, which will allow for the extension of cultivable lands or increase the productivity of established agricultural soils and thus increase food production and diminish the water footprint. This is especially urgent in drought-prone areas devoted to the production of horticultural crops, such as the Mediterranean area, California, Florida, or South Africa, among many others. Predicted climate change is expected to exacerbate the negative impact of extreme drought events;^3^ moreover, drought is one of the main factors driving deforestation in developing countries.^4^

There is considerable knowledge regarding the physiology, biochemistry, and molecular genetics of plants under drought stress, but applying this knowledge to produce new cultivars of crops able to maintain yield under drought stress or adapt to arid environments has proven to be very difficult.^5^ Genetic engineering has shown limited success thus far. Although there are many descriptions in the literature of drought-tolerant crops by means of genetic engineering,^6^ there are only two cultivars in the market whose trait conferred by the transgene is drought tolerance: the DroughtGard maize^7^ and very recently the soybean expressing the HB4 transcription factor from sunflower.^8^ The problem is that in most cases, the strategy is a bottom-up approach starting at the molecular level, and thus the selection of the transgene is based on evidence provided by results in other plants or data base mining of results of gene expression during abiotic stress. This approach may fail as it does not detect the limiting factor(s) or because significant tolerance in the field is mediated by many genes with additive effects. To avoid this problem, several experimental designs have proven to be effective, such as screening for genes in heterologous systems.^9,10^ However, to take advantage of this knowledge, new GMO crops must be developed, which is still a problem to market in many countries and requires a long and expensive process of regulation prior to approval.

Here, we present an alternative top-down strategy to use field experience to identify tolerance markers at the molecular level.^2^ Broccoli (*Brassica oleracea L*. var. *Italica* Plenck) is a major horticultural crop, cultivated in temperate areas. In 2018, the world production of broccoli and cauliflower was about 25 million tonnes, with China and India as the main producers worldwide, accounting for 70% of the total production. USA is the main producer in the Americas, and Spain is the top producer in Europe (data available in FAO sums up broccoli and cauliflower production).^11^ Broccoli is also a source of dietary health-protecting molecules.^12−14^ Most of these molecules are resistant to standard cooking techniques;^15^ therefore, broccoli is recommended in most diets. Maintaining the yield of this highly nutritional crop and diminishing the environmental impact on its production are the objectives of most breeders. To attain these objectives, it is necessary to increase its tolerance to drought stress or at least decrease the water footprint^16^ of broccoli production.

The comparison of the physiological and molecular responses among drought-tolerant and drought-sensitive populations to identify differential traits has proven to be a useful strategy to characterize the abiotic response of specific crops^17,18^ or even forest species.^19,20^ To apply this strategy to broccoli, we have identified drought-tolerant and drought-sensitive precommercial cultivars of this crop based on field and greenhouse experiments. Then, we have characterized the physiological and molecular responses of these different broccoli cultivars under controlled drought stress, aiming at identifying distinctive traits for drought tolerance. Studies at the molecular level require the use of controlled greenhouse conditions, given that in the field, there are many variables (such as the presence of pathogens, different light exposure, wound stress caused by strong wind, rain, or insect attack, or mechanical stimulation) that can differentially affect plants and therefore generate excess variability in the results. The data generated in the current study may be useful to predict whether broccoli cultivars that have not been tested in field trials will be suitable for planting in drought-prone areas and to breed novel cultivars with increased amounts of metabolites or physiological traits that are limiting under drought stress. Therefore, this approach constitutes a top-down-top strategy because the final objective is to transfer the knowledge acquired in the laboratory to the field by determining which cultivars are going to be more resistant to drought stress based on their physiological, biochemical, or metabolomic profile.

## Materials and Methods

### Plant Material and Treatments

This study was performed using four broccoli precommercial cultivars provided by SAKATA. Cultivars were preselected among 12 precommercial varieties based on their survival and fitness under drought conditions. We confirmed the reproducibility of the results employing controlled drought stress greenhouse experiments (Supporting Information, Figure S1). Plants were grown following common procedures reported in the literature for this species. Greenhouse conditions were as follows: 16 h light/8 h dark (200 μmol m^–2^ s^–1^ of light intensity) at 24 ± 2 °C and 70 ± 5% relative humidity in pots containing a 1:2 vermiculite/soil mixture arranged in a complete random block design with six blocks, where the different seed sources were randomized within the block. Plants were watered to full capacity every 2 days with complete Hoagland’s nutrient solution^21^ containing all essential macronutrients and micronutrients as described previously.^22^ After 5 weeks of growth, healthy plants of similar size from each cultivar, accounting for five replicates per cultivar and treatment, were randomly assigned to control and drought treatments. Control plants were irrigated every 2 days, whereas drought conditions were applied by withholding water until the total weight (plant and container) was reduced to 60% of their initial weight. To obtain the florets, plants were grown for 3 months under greenhouse conditions (16 h light/8 h dark) (200 μmol m^–2^ s^–1^ of light intensity) at 24 ± 2 °C and 70 ± 5% relative humidity in individual plant pots (25 cm diameter, 25 cm height). Once florets were developed, a drought treatment was applied through withholding water until the seedling weight (plant and container) was reduced to 60% of their initial weight, while control plants were kept with normal watering.

### Physiological Measurements

Measurements were performed in the third youngest leaf according to previous studies.^19^ The water potential (Ψw, MPa) was measured with a Schölander pressure pump (model PMS-1000, PMS Instruments, Corvallis, OR) in five plants of each cultivar and treatment. A CIRAS-3 portable photosynthesis system (PP Systems, Amesbury MA) was used for gas exchange determinations. The conditions were saturating light (1500 μmol of photons m^–2^ s^–1^) with a temperature of 25 °C, controlled ambient CO_2_ (390 μmol mol^–1^ CO_2_), and a relative humidity of approximately 55%. The instantaneous determination of net CO_2_ assimilation photosynthesis (A, μmol CO_2_ m^–2^ s^–1^), stomatal conductance (gs, mol m^–2^ s^–1^), transpiration (E, mmol H_2_O^2–^ s^–1^), and instantaneous water use efficiency (WUE, μmol CO_2_ mmol^–1^ H_2_O), which is defined by the relationship between photosynthesis and stomatal conductance, were determined in the same leaves in five replicates for each cultivar.

### Amino Acid Analysis

Glutathione (GSH) and free amino acids were extracted from 0.1 g of lyophilized leaves according to the method described previously.^20^ In brief, plant material was pooled and homogenized using a mortar and pestle. Each pooled sample (0.10 g of dry weight) was heated for 12 min at 95 °C in 2% isocitrate buffer (pH 2 with HCl).^23^ In all, 1–10 dilutions of these extractions were injected in a Beckman Gold amino acid automatic analyzer. The analysis was carried out following the protocol provided by the manufacturer, using a sodium citrate system and ninhydrin for detection as described previously.^24^

### Ion Content Determination

Ions were determined as described previously.^25^ Briefly, samples of the third youngest leaf from the indicated plants (about 1 g) were lyophilized for 3 days. Dry weight was determined, and ions were extracted by a 30 min incubation in 1 mL of 0.1 M HNO_3_ at room temperature. Then, samples were centrifuged, and the supernatant was diluted with 4 mL of milli-Q water and filtered (0.22 μM). Sodium and potassium were measured in a plasma emission spectrophotometer (Shimadzu), as described previously.^26^

### Hormone Determinations

Abscisic acid (ABA), salicylic acid (SA), jasmonic acid (JA), and indoleacetic acid (IAA) were quantified as described previously.^27^ A freeze-dried lyophilized tissue from the third youngest leaf (50 mg) was extracted with 2 mL of water after spiking with ^[2^H_6_]-ABA, [^2^H_3_]-PA, dehydrojasmonic acid (DHJA), and [^13^C]-SA applying mechanical stress with a ball mill (MillMix20, Domel, Zelezniki, Slovenia). Extracts were centrifuged (4000*g*, 10 min, 4 °C), supernatants were collected, and the pH was adjusted to 3 with acetic acid. The extract was partitioned twice against diethyl ether. The upper layer was collected and evaporated (Speed Vac, Jouan, Saint Herblain Cedex, France). The dry residue was resuspended in 10% MeOH, sonicated, filtered (0.22 μM, Albet S.A., Barcelona, Spain), and injected into an UPLC system (Acquity SDS, Waters Corp., Milford, MA). Analytes were separated using a reversed-phase C18 column (Gravity, 1.8 μm, 50 × 2.1 mm, Macherey-Nagel, Düren, Germany) using a 300 μL min^–1^ linear gradient of ultrapure H_2_O (A) and MeOH (B) (both supplemented with 0.01% acetic acid). The gradient was: (0–2 min) 90:10 (A/B), (2–6 min) 10:90 (A/B), and (6–7 min) 90:10 (A/B). For quantification, we used a Quattro LC triple quadrupole mass spectrometer (Micromass, Manchester, U.K.) connected online to the output of the column through an orthogonal Z-spray electrospray ion source. Quantitation of hormones was achieved based on a standard curve. Three biological replicates per cultivar and treatment were analyzed for each sampling time, and each sample was measured twice.

### Metabolomic Analysis

The third youngest leaf was collected and lyophilized and then homogenized with a mechanical tissue disruptor in the presence of liquid nitrogen before obtaining 10 mg of sample powder for each replicate. Four biological replicates of each cultivar and treatment were analyzed using a method modified from ref (28). Powder was extracted in 1.4 mL of 100% methanol and 60 μL of an internal standard (0.2 mg of ribitol in 1 mL of water). The mixture was heated for 15 min at 70 °C and centrifuged (10 min; 20 000*g*). The supernatant was transferred to a glass vial, and then 750 μL of chloromethane and 1.5 mL of water were added. The mixture was vortexed for 15 s and centrifuged for 15 min at 20 000*g*. Aliquots (150 μL) of the methanol/water supernatant were dried by evaporation for 6–16 h.

For derivatization, dry residues were dissolved in 40 μL of 20 mg/mL methoxyamine hydrochloride in pyridine and incubated for 90 min at 37 °C, followed by addition of 70 μL of *N*-methyl-*N*-[trimethylsilyl]trifluoroacetamide (MSTFA) and 6 μL of a retention time standard mixture (3.7% (w/v) mixture of fatty acid methyl esters ranging from 8 to 24 °C) and further incubation for 30 min at 37 °C.

Sample volumes of 2 μL were injected in split and splitless mode to increase the metabolite detection range in a 6890 N gas chromatograph (Agilent Technologies Inc., Santa Clara, CA) coupled to a Pegasus 4D TOF mass spectrometer (LECO, St. Joseph, MI). Gas chromatography was performed on a BPX35 (30 m × 0.32 mm × 0.25 μm) column (SGE Analytical Science Pty Ltd., Australia) with helium as the carrier gas at a constant flow of 2 mL/min. The liner was set at 230 °C. The oven program was set at 85 °C for 2 min, and then the temperature was increased with an 8 °C/min ramp until 360 °C. Mass spectra were collected at 6.25 spectra s^–1^ in the *m*/*z* range 70–800 and ionization energy of 70 eV. Chromatograms and mass spectra were evaluated using the CHROMATOF program (LECO, St. Joseph, MI).

### Statistical Analysis

Analysis of variance was carried out to determine significant differences between means at a *p* < 0.05 level. Homogeneous groups were separated using the Duncan multiple range test (MRT). In all cases, data were examined for normality and homogeneity of variances and assessed for any violations of assumptions. The data analysis for this project was generated using SPSS software (IBM SPSS Statistics for Windows, Version 22.0. Armonk, NY).

## Results

### Physiological Measurements

**Figure 1:**
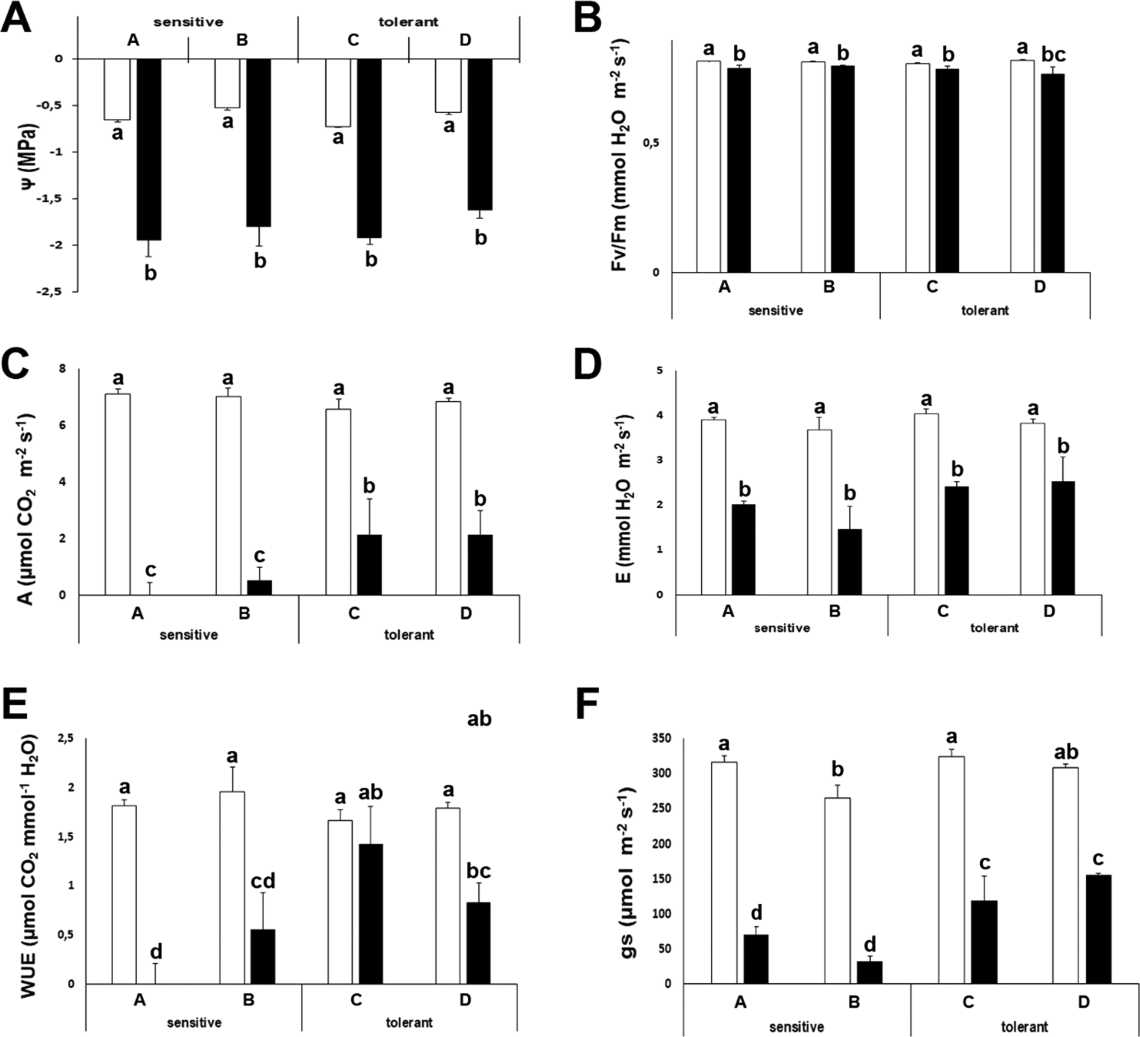
Physiological measurements. Water potential (Ψw) (A), maximal efficiency of PSII (Fv/Fm) (B), net photosynthesis (A) (C), transpiration (E) (D), instantaneous water use efficiency (WUE) (E), and stomatal conductance (gs) (F) of drought-sensitive and drought-tolerant cultivars under watered (white bars) and drought-stressed (black bars) treatments. Data with different letters differ significantly (*p* < 0.05), as determined by Duncan’s MRT (*n* = 5). Scale bars are mean + statistical error (SE).

The first aim of our study was to test the physiological response of the plants (visual fitness upon irrigation withdrawal, fresh weight and drought weight ratio under control and stress conditions, and ratio of stress/control conditions) and to check the stress conditions to validate the experimental design (Supporting Information, Figure S1). To check the effect of the selected drought stress conditions, we determined several functional parameters. Under drought stress, water potential decreased significantly (about fourfold), indicating that the plants were indeed experiencing drought stress. The same response was observed for the other parameters, such as maximal PSII efficiency (Fv/Fm) and transpiration (E). For photosynthetic rates (A), WUE, and stomatal conductance (gs), the drought-tolerant cultivars exhibited a significant improvement when compared to the drought-sensitive cultivars (Figure 1).

### Glutathione and Free Amino Acids

**Figure 2:**
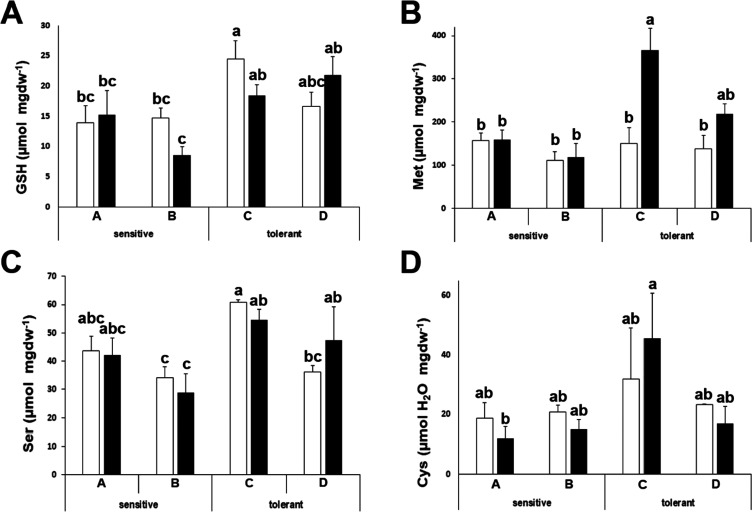
Glutathione, sulfur-containing amino acids, and serine determination. Glutathione (GSH) (A), methionine (Met) (B), serine (Ser) (C), and cysteine (Cys) (D) concentrations of drought-sensitive and drought-tolerant cultivars under watered (white bars) and drought-stressed (black bars) conditions. Data with different letters differ significantly (*p* < 0.05), as determined by Duncan’s MRT (*n* = 3). Scale bars are mean + SE.

Drought stress induces oxidation, and under these conditions, the biosynthesis of sulfur-containing amino acids may become limiting due to the requirement of cysteine for the biosynthesis of glutathione (GSH).^29^ In our study, GSH accumulated to higher levels in drought-tolerant plants, independently of the stress (Figure 2A). The levels of methionine (Met) were higher in drought-tolerant plants after stress (Figure 2B), while the levels of serine (Ser), which is required for cysteine and methionine biosynthesis, showed a similar pattern to GSH (Figure 2C). We did not find a distinctive pattern for cysteine (Figure 2D).

**Figure 3:**
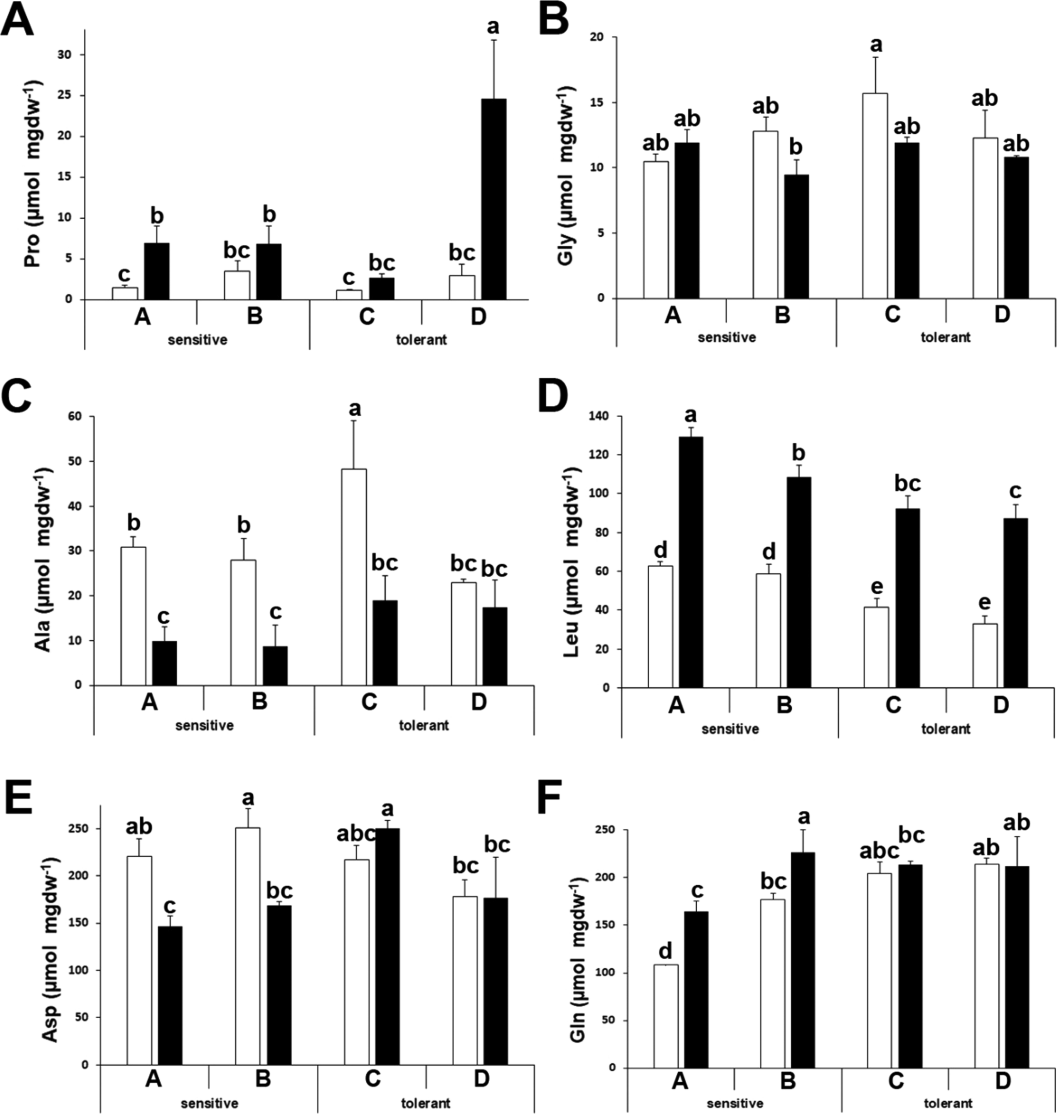
Amino acids that can act as osmolytes or with differential patterns between stress-sensitive and stress-tolerant cultivars. Proline (Pro) (A), glycine (Gly) (B), alanine (Ala) (C), leucine (Leu) (D), aspartic acid (Asp) (E), and glutamine (Gln) (F) concentrations of drought-sensitive and drought-tolerant cultivars under watered (white bars) and drought-stressed (black bars) conditions. Data with different letters differ significantly (*p* < 0.05), as determined by Duncan’s MRT (*n* = 3). Scale bars are mean + SE.

We further investigated the levels of free amino acids. Some amino acids can act as precursors for osmolytes, act as osmolytes themselves, or even could have previously undescribed functions in stress tolerance. For instance, proline (Pro) and glycine (Gly) are related to osmotic adjustment and can act as osmolytes or, in the case of proline, even as an antioxidant.^30^ As expected, proline accumulated under drought stress, although the magnitude of this change did not correlate with the tolerance to drought stress (Figure 3A). Glycine concentrations showed a slight and nonsignificant decrease upon stress, and levels were similar for sensitive and tolerant cultivars (Figure 3B).

We examined the patterns of the others amino acids looking for differences between sensitive and tolerant cultivars. The alanine (Ala) concentration decreased in stressed plants, but tolerant cultivars maintained a higher level (Figure 3C). Leucine (Leu), also a hydrophobic amino acid, showed the opposite pattern, as levels increased after stress, but concentrations were lower for tolerant cultivars independently of the treatment (Figure 3D). Aspartic acid (Asp) levels changed divergently among sensitive (decrease) and tolerant (increase or maintain) cultivars (Figure 3E). A similar effect was found for glutamine (Gln), which increased upon stress in sensitive cultivars, while tolerant cultivars presented higher levels that were maintained in stressed plants (Figure 3F).

**Figure 4:**
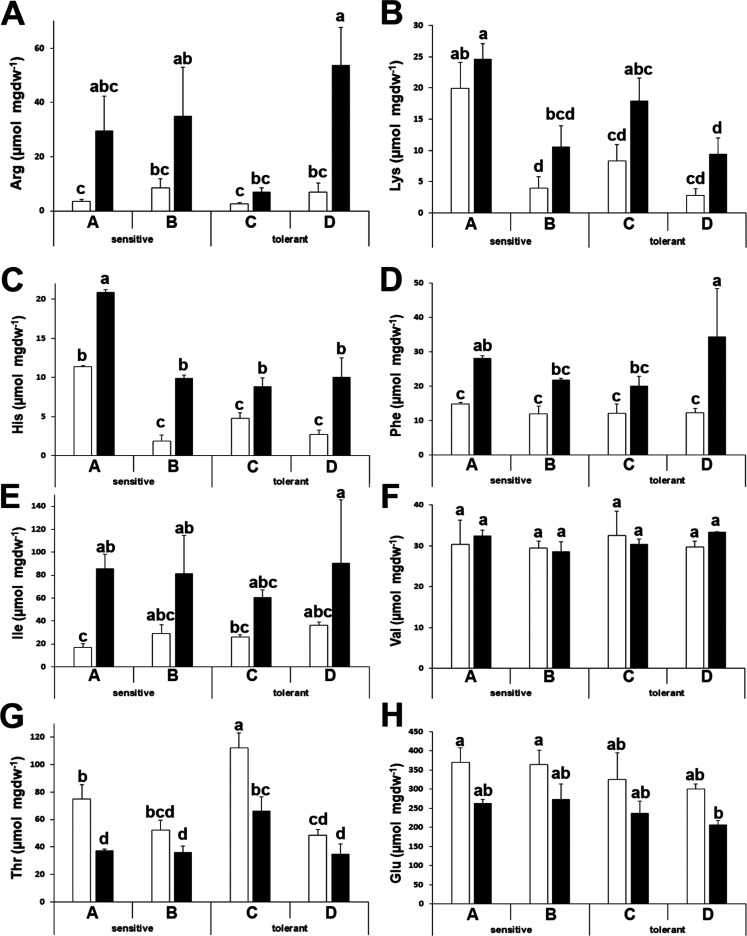
Amino acids with similar patterns between stress-sensitive and stress-tolerant cultivars. Arginine (Arg) (A), lysine (Lys) (B), histidine (His) (C), phenylalanine (Phe) (D), isoleucine (Ile) (E), valine (Val) (F), threonine (Thr) (G), and glutamic acid (Glu) (H) concentrations of drought-sensitive and drought-tolerant seed sources under watered (white bars) and drought-stressed (black bars) treatments. Data with different letters differ significantly (*p* < 0.05), as determined by Duncan’s MRT (*n* = 3). Scale bars are mean + SE.

For some other amino acids, we did not observe differences among sensitive and tolerant cultivars, but there is no description available in the literature regarding the behavior of the pools of the free proteinic amino acids under drought stress in broccoli. Arginine (Arg), lysine (Lys), histidine (His), phenylalanine (Phe), and isoleucine (Ile) showed increased levels after stress (Figure 4A–E), whereas valine levels were stable and unaffected by stress (Figure 4F). Threonine (Thr) and glutamic acid (Glu) levels decreased after stress (Figure 4G,H).

### Concentration of Essential Amino Acids in Florets

**Figure 5:**
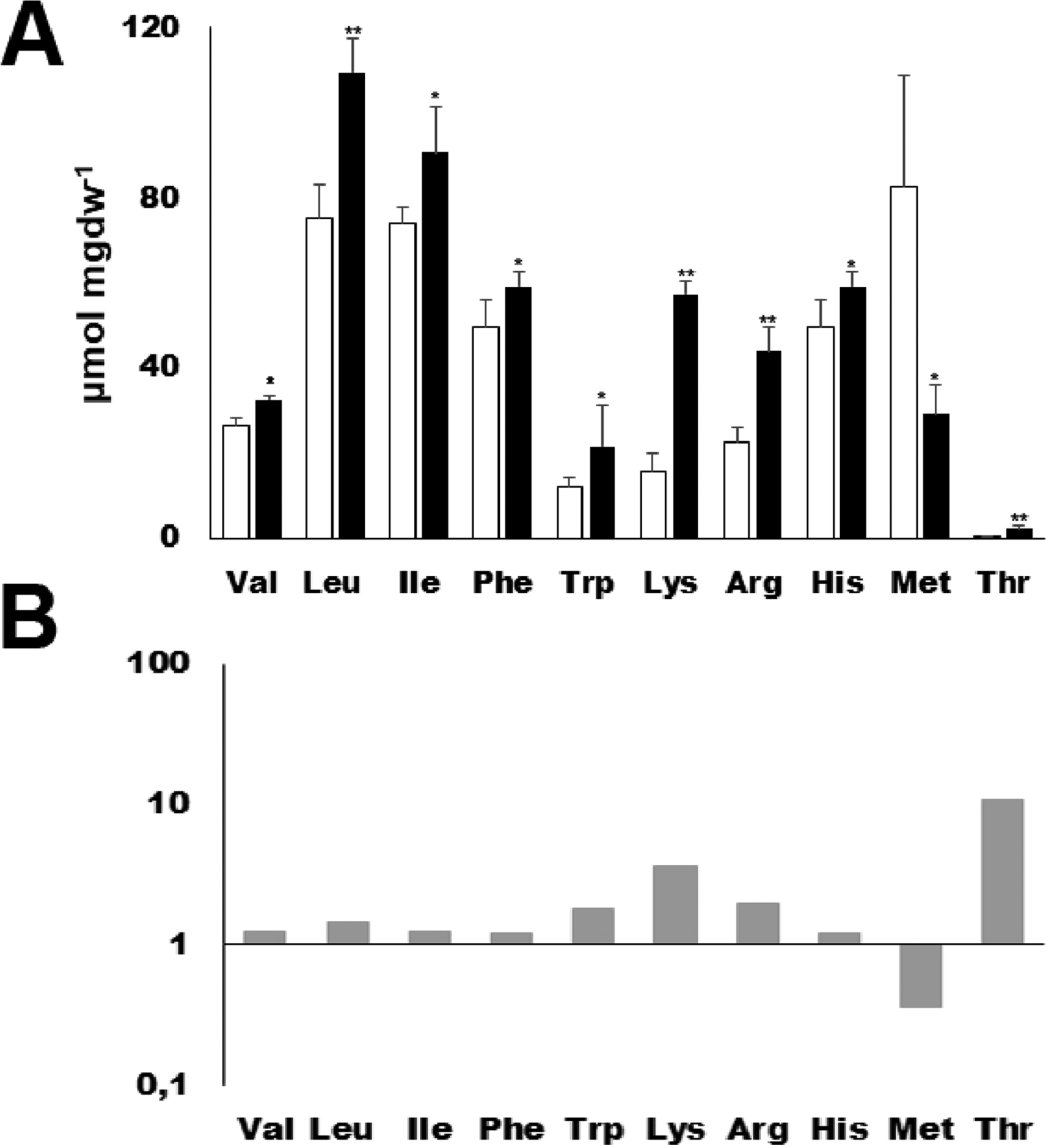
Determination of essential amino acids in florets. Concentrations of the indicated amino acids in florets under watered (white bars) and drought-stressed (black bars) treatments (A) and the ratio of the stress/control concentrations (B). Asterisks indicate the *p* values (* = *p* < 0.01 and ** = *p* < 0.001), as determined by Student’s *t*-test (*n* = 3). Scale bars are the mean + SE.

Analyzing the results from amino acid determinations (Figures 3 and 4), we noticed that drought increases the concentration of amino acids essential for the human diet. However, these concentrations were measured in leaves from 5-week-old plants rather than the edible part of broccoli (florets). This opens the possibility that a drought treatment could increase the content of essential amino acids in broccoli and thus increase its nutritional value. To test this hypothesis, we cultivated a different cultivar until the development of the bud, and we applied the drought stress and determined the concentrations of essential amino acids in florets. We used a different cultivar to confirm that we were observing a general pattern for broccoli independent of the cultivar (Figure 5A). We could observe an increase in all essential amino acids, except for Met. Increases ranged from 1.2- to 10-fold (Figure 5B).

### Sodium and Potassium Content

**Figure 6:**
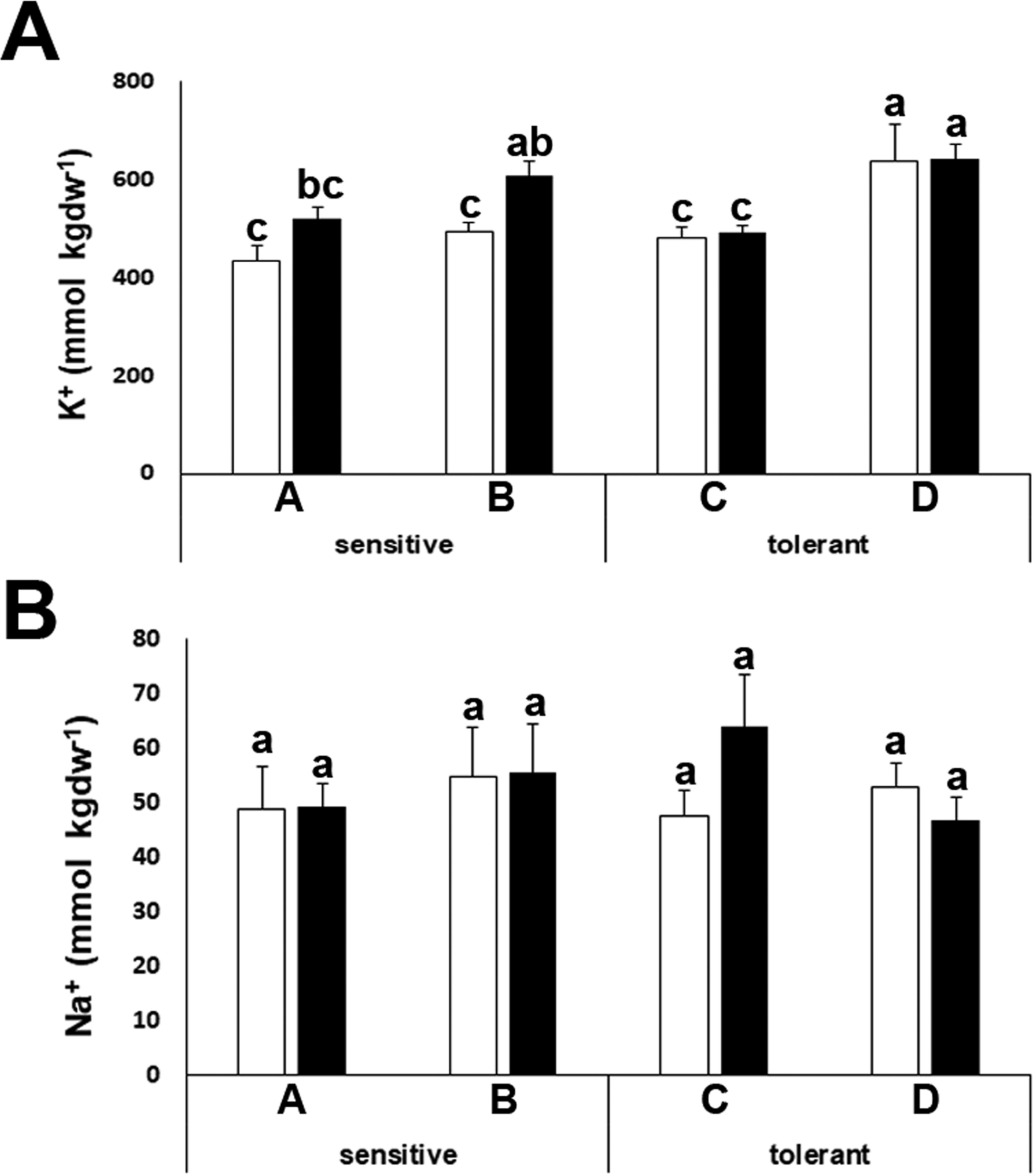
Ion content determination. Potassium (K^+^) (A) and sodium (Na^+^) (B) concentrations of drought-sensitive and drought-tolerant cultivars under watered (white bars) and drought-stressed (black bars) treatments. Data with different letters differ significantly (*p* < 0.05), as determined by Duncan’s MRT (*n* = 8). Scale bars are mean + SE.

Plant cells must maintain a stable ionic environment under a range of external conditions. Potassium is the major ion in the internal medium of plants; the concentration in the cell cytoplasm must be stable and about 150 mM, independently of the external concentration. At the same time, sodium, an abundant cation in most soils, must be maintained outside the cytoplasm due to its toxicity. In addition, potassium fluxes are determinants of basic cellular processes involved in the drought stress response, such as stomatal aperture.^31^ Potassium can also act as an osmolyte under stress conditions. We investigated whether the potassium content in leaves could be a distinctive feature for drought tolerance in broccoli. We determined sodium and potassium content in leaves under control and stress conditions. We observed only minor differences among cultivars and treatments for both cations, suggesting that potassium homeostasis is not a limiting factor for drought tolerance in the cultivars analyzed (Figure 6).

### Hormone Determinations

**Figure 7:**
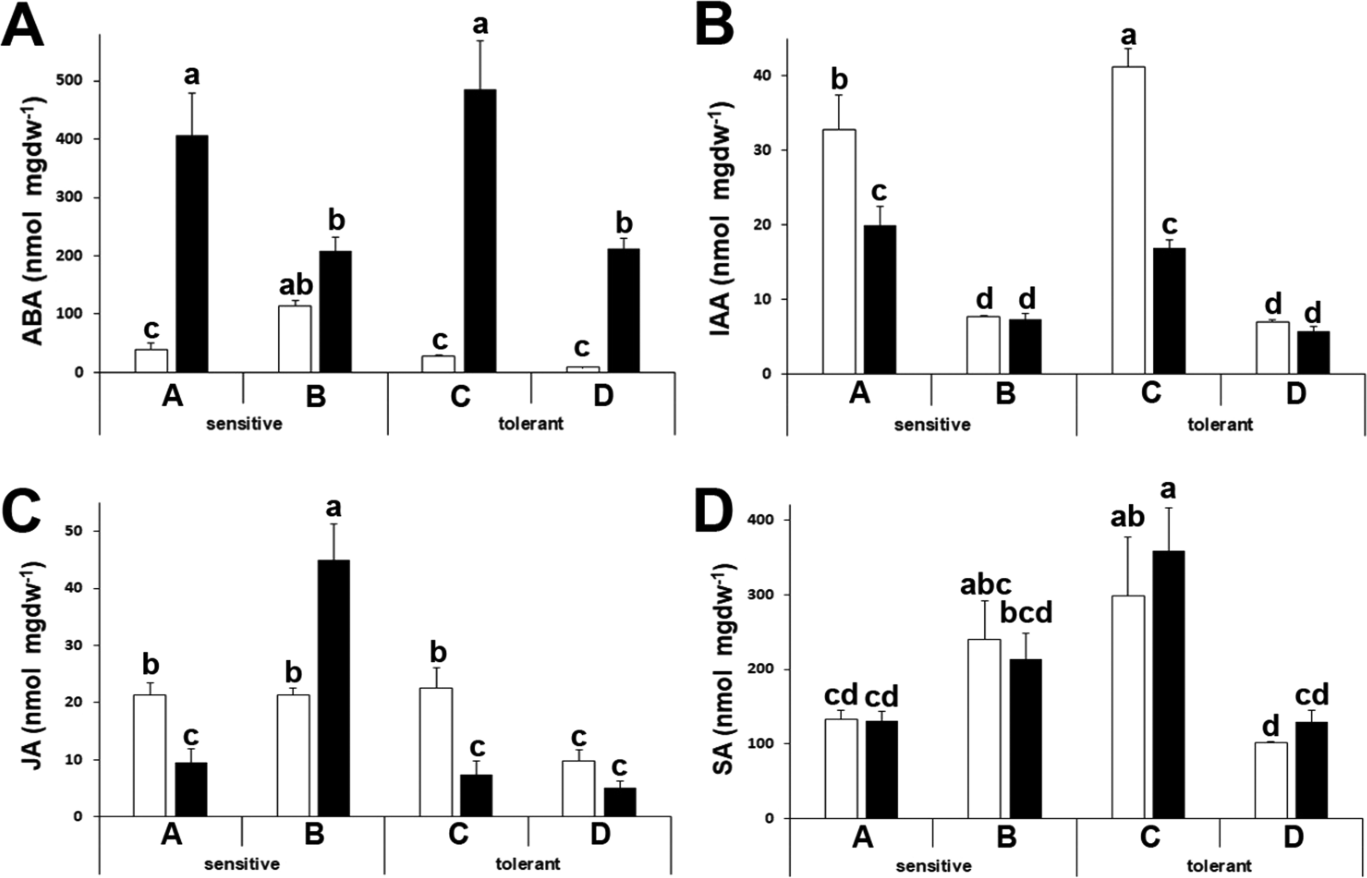
Hormone concentrations. Abscisic acid (ABA) (A), indolacetic acid (IAA) (B), jasmonic acid (JA) (C), and salicylic acid (SA) (D) concentrations of drought-sensitive and drought-tolerant cultivars under watered (white bars) and drought-stressed (black bars) conditions. Data with different letters differ significantly (*p* < 0.05), as determined by Duncan’s MRT (*n* = 6). Scale bars are mean + SE.

Hormones play a major role in stress responses as they are responsible for transducing the signal to the whole plant. Abscisic acid (ABA) is the main hormone responsible for the abiotic stress response, and, as expected, its levels increased upon stress (Figure 7A). The relative increase was higher in drought-tolerant cultivars (Figure 7A). Auxin (IAA) and jasmonic acid (JA) concentrations decreased upon stress, but we did not observe a distinctive pattern (Figure 7B,C). Salicylic acid (SA) has been described as being able to increase the tolerance to abiotic stress in horticultural crops when applied exogenously.^18^ We found that levels of SA were stable upon stress. Levels diverged about 2- to 3-fold between cultivars, but again, we did not observe a distinctive pattern between drought-tolerant and drought-sensitive cultivars (Figure 7D).

### Primary Metabolite Analysis

**Figure 8:**
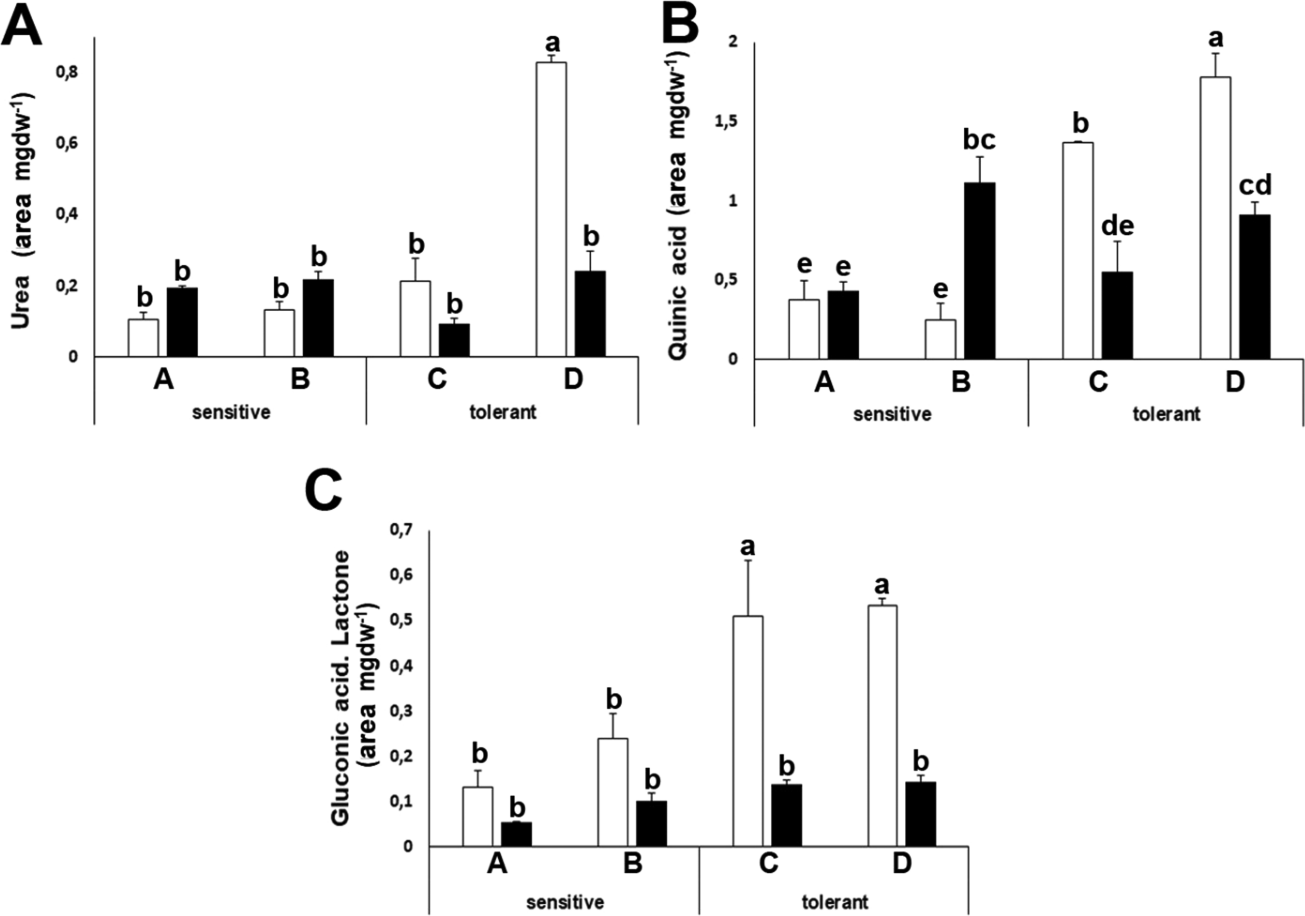
Concentrations of primary metabolites with differential patterns among cultivars. Urea (A), quinic acid (B), and gluconic acid lactone (C) of drought-sensitive and drought-tolerant cultivars under watered (white bars) and drought-stressed (black bars) conditions. The units are the area of the peak per mg of sample. Data with different letters differ significantly (*p* < 0.05), as determined by Duncan’s MRT (*n* = 4). Scale bars are the mean + SE.

Finally, we investigated whether we could identify distinctive traits among the cultivars by analyzing primary metabolites. We did not observe any distinctive pattern when we analyzed sugars or intermediates of the Krebs cycle (data not shown), but we found that urea and quinic acid levels increased upon stress in drought-sensitive cultivars, while decreasing in the drought-tolerant ones (Figure 8A,B). Also, the levels of the lactone of gluconic acid were higher for drought-tolerant cultivars under control conditions (Figure 8C).

## Discussion

We designed this study to find limiting factors for drought stress tolerance in broccoli using a molecular and physiological approach. The identified differential traits could be useful for breeding new cultivars of broccoli with less water requirement. The main findings are summarized in Supporting Information Figure S2.

Throughout this study, the well-established marker of drought stress, water potential (Ψw), and the known drought stress response molecules, Pro and ABA, exhibited the expected differences with respect to control plants. These results validate our experimental design and confirm that the plants in the greenhouse were affected by drought stress. Interestingly, from these three parameters, we only found a differential response between tolerant and sensitive cultivars for ABA, pointing out that the amount of this hormone may be limiting under drought stress conditions. Potassium can also act as an osmolyte, but we have shown here that it is not the limiting factor for drought stress tolerance, as we did not find significant differences among tolerant and sensitive cultivars nor a significant increase upon drought stress. A known strategy of plants during drought stress is to downregulate the energy status to avoid oxidation.^19,32,33^ We have observed this downregulation in broccoli (Figure 1B). At the physiological level, gs, A, and WUE differ significantly among cultivars, indicating that they could be used as markers for stress tolerance.

It is known that one of the main problems caused by drought is oxidative damage.^34^ The biosynthesis of cysteine from serine, and specifically the activity of the serine *O*-acetyltransferase,^29,35^ is a known limiting step for abiotic stress tolerance. Several reports indicate that GSH is considered to be the most important thiol involved in the prevention of oxidative damage in plants.^36,37^ In addition, broccoli is rich in sulfur-containing molecules, such as glucosinolates. It has been suggested that the levels of GSH (required for the biosynthesis of glucosinolates) and Met (one of the main precursors) are important to maintain the biosynthesis of pivotal molecules for the defense against herbivores in broccoli.^38^ We could see that the levels of GSH, Met, and Ser in leaves were higher for drought-tolerant plants, indicating that in broccoli, sulfur metabolism is also a limiting factor for drought stress tolerance and that the antioxidant response involving glutathione or sulfur-containing proteins is a limiting factor for tolerance (Figure 2).

We have mentioned before that the ABA concentration is a limiting factor for drought tolerance. Hormone responses are as expected for broccoli under abiotic stress,^39^ but the levels of IAA, JA, or SA are not a distinctive trait in our cultivars, as hormonal levels did not correlate with tolerance (Figure 7). Also, we have found that urea and quinic acid decreased in drought-tolerant cultivars (Figure 8). In our experimental conditions, all plants had the same level of nitrogen fertilization, so observed changes do not represent changes in nitrogen uptake from the soil but in nitrogen metabolism. The main source of urea is the degradation of arginine.^40^ We have found that arginine accumulates upon drought stress (Figure 4A), so the observed decrease in urea could be explained by an inhibition of the arginase activity, the enzyme that converts arginine into urea. In addition to urea, Arg is the immediate precursor of several molecules related to stress responses, such as nitric oxide (NO), ornithine, and agmatine,^41,42^ and is also the precursor of creatine, polyamines, and glutamate.^43^ External application of Arg has also been shown to alleviate drought stress in some crops.^44^ Therefore, Arg might play a crucial role in stress recovery, and a decrease in its turnover to urea may be a marker for drought stress tolerance. We also found a distinctive pattern for quinic acid and the gluconic acid lactone, although its biochemical interpretation is not obvious. We can summarize these results stating that we have found that high levels of Met and ABA, together with low levels of urea, quinic acid, and gluconic acid lactone, constitute a signature for drought tolerance in broccoli.

The strategy that we have used has another interesting advantage. It is known that stress can increase the organoleptical^45^ or health-promoting properties^46^ of several crops. So, studying the chemical profile of broccoli during drought stress could be a useful tool, not only for maintaining yield under adverse conditions but also to describe conditions in which its nutritional content may increase. Water stress has been shown to delay postharvest yellowing in broccoli florets.^47^ Here, we show that a drought treatment increases the content of all essential amino acids (except Met) in the edible part of broccoli (Figure 5). There are previous descriptions in the literature of treatments that can be applied to broccoli that could enhance its nutritional content or delay its senescence.^48,49^ Although broccoli is not considered a rich protein source, we have observed that drought treatment enhances its nutritional content. It is interesting to note that Met increases in leaves but decreases in florets. As mentioned before, Met is the precursor of glucosinolates and other molecules related to stress defense, such as polyamines. It is likely that the biosynthesis of these molecules under stress is more of a determinant in leaves. Taken all together, we have identified several limiting factors for broccoli tolerance to drought stress, which could define novel targets for breeding programs or for the biotechnological improvement of broccoli, aiming at creating novel cultivars adapted to drought-prone areas.

## Supporting Information Available

Representative plants of each cultivar under normal watering (upper panel) or after 6 days of drought stress (lower panel) (Figure S1) and summary of the main findings of this study: radial diagrams of the ratio between stress and control concentrations (A) and control and stress concentrations (B); values are represented on a decimal logarithmic scale (Figure S2) (PDF)
