# Supplementary material for: Physiological and Molecular Characterization of the Differential Response of Broccoli (Brassica oleracea var. Italica) Cultivars Reveals Limiting Factors for Broccoli Tolerance to Drought Stress
Source: J Agric Food Chem. 2021 Aug 27;69(35):10394–404. doi: 10.1021/acs.jafc.1c03421 (PMC8528380; doi:10.1021/acs.jafc.1c03421)
Supplement: Supplementary file 1 — jf1c03421_si_001.pdf [file jf1c03421_si_001.pdf]

SUPPLEMENTAL FIGURE 1. Representative plants of each cultivar under normal watering (upper panel) or after 6 days of drought stress (lower panel) (A); The third leave of each plant was cut and fresh weight and dry weight was determined of drought-sensitive and drought-tolerant cultivars under watered (white bars) and drought-stress (black bars) treatments (upper panel) the ratio between stress and control conditions (lower panel) (B). Data with different letters differ significantly ($p < 0.05$), as determined by Duncan's MRT test ($n = 5$). Scale bars are mean + Statistical Error (SE).

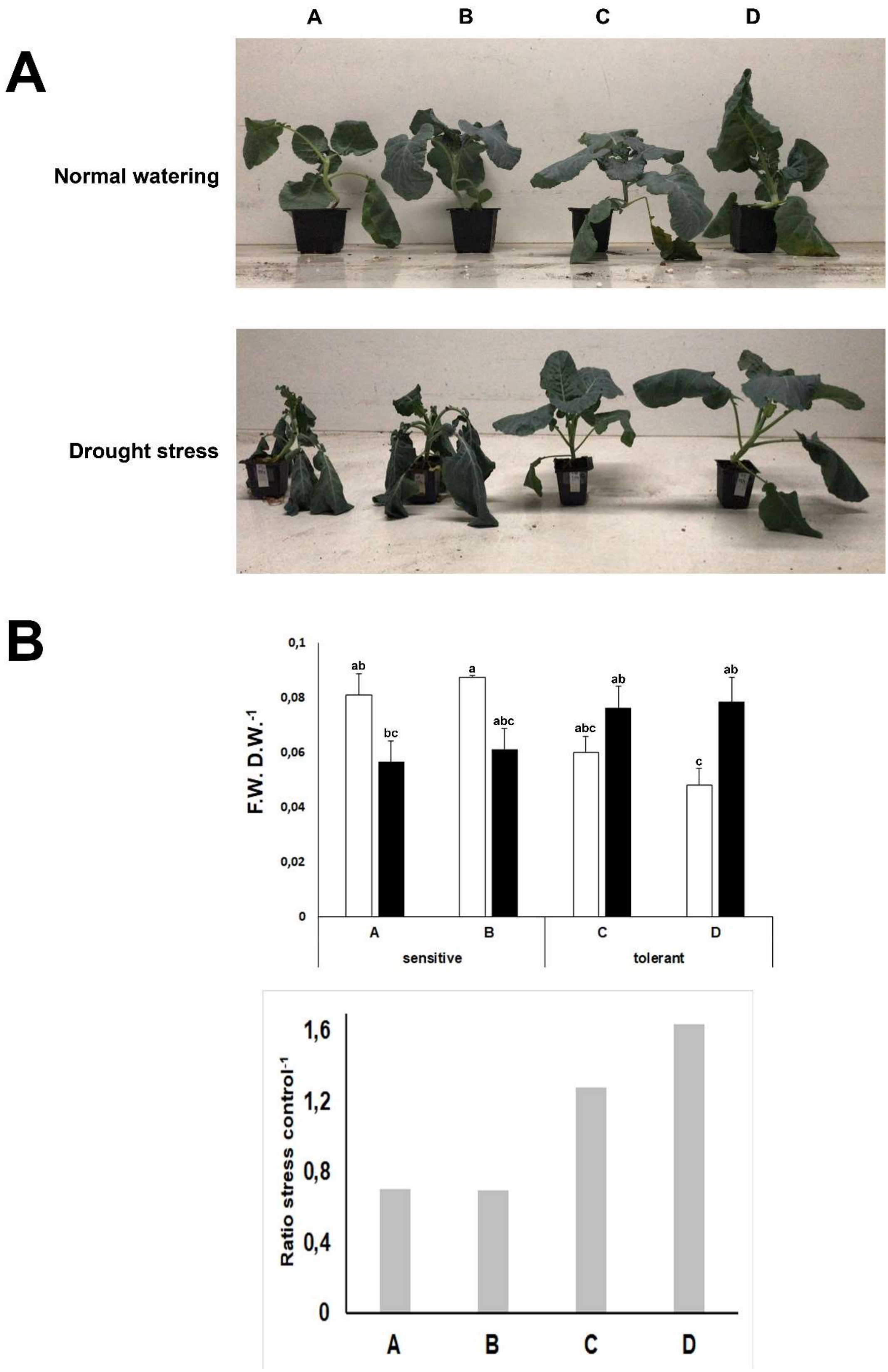

A
B
C
D
A
Normal watering
Drought stress
B
0,1
0,08
0,06
0,04
0,02
0
F.W. D.W.-1
ab
bc
a
abc
abc
ab
c
ab
A
B
C
D
sensitive
tolerant
Ratio stress control-1
1,6
1,2
0,8
0,4
0
A
B
C
D

SUPPLEMENTAL FIGURE 2: Summary of the main findings of this study. Radial diagrams of the ratio between stress/control concentrations (A) and control/stress concentrations (B). Values are represented in a decimal logarithmic scale.

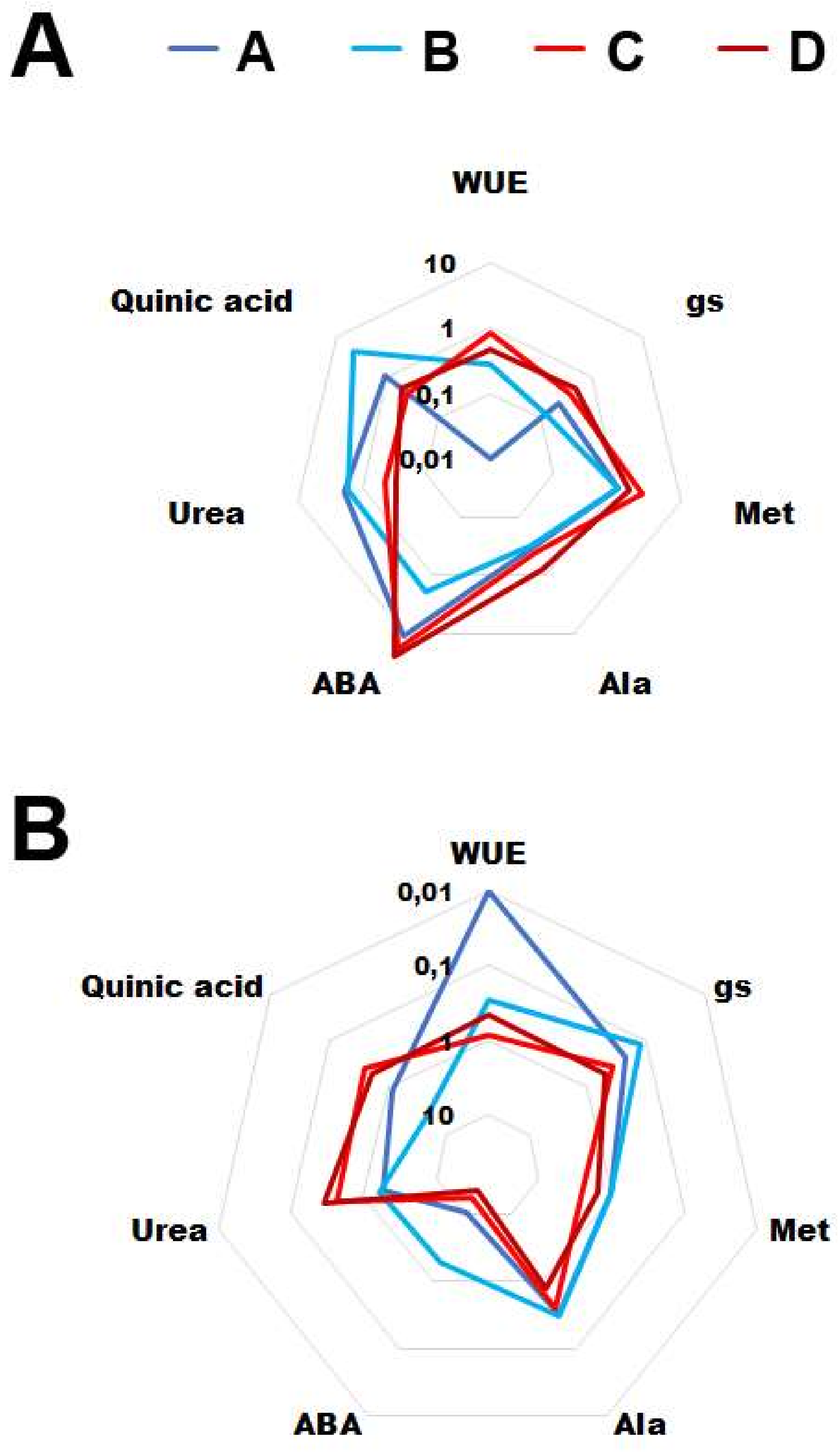
A
A
B
C
D
WUE
gs
Met
Ala
ABA
Urea
Quinic acid
10
1
0,1
0,01
B
WUE
gs
Met
Ala
ABA
Urea
Quinic acid
0,01
0,1
1
10
